# Saudi Arabian Y-Chromosome diversity and its relationship with nearby regions

**DOI:** 10.1186/1471-2156-10-59

**Published:** 2009-09-22

**Authors:** Khaled K Abu-Amero, Ali Hellani, Ana M González, Jose M Larruga, Vicente M Cabrera, Peter A Underhill

**Affiliations:** 1 Molecular Genetics Laboratory, College of Medicine, King Saud University, Riyadh 11411, Saudi Arabia; 2 Department of PGD, Saad Specialist Hospital, Al-Khobar, Saudi Arabia; 3 Departamento de Genética, Universidad de La Laguna, 38271 La Laguna, Tenerife, Spain; 4 Department of Psychiatry and Behavioural Sciences, Stanford University, School of Medicine, Stanford, California 94304, USA

## Abstract

**Background:**

Human origins and migration models proposing the Horn of Africa as a prehistoric exit route to Asia have stimulated molecular genetic studies in the region using uniparental loci. However, from a Y-chromosome perspective, Saudi Arabia, the largest country of the region, has not yet been surveyed. To address this gap, a sample of 157 Saudi males was analyzed at high resolution using 67 Y-chromosome binary markers. In addition, haplotypic diversity for its most prominent J1-M267 lineage was estimated using a set of 17 Y-specific STR loci.

**Results:**

Saudi Arabia differentiates from other Arabian Peninsula countries by a higher presence of J2-M172 lineages. It is significantly different from Yemen mainly due to a comparative reduction of sub-Saharan Africa E1-M123 and Levantine J1-M267 male lineages. Around 14% of the Saudi Arabia Y-chromosome pool is typical of African biogeographic ancestry, 17% arrived to the area from the East across Iran, while the remainder 69% could be considered of direct or indirect Levantine ascription. Interestingly, basal E-M96* (n = 2) and J-M304* (n = 3) lineages have been detected, for the first time, in the Arabian Peninsula. Coalescence time for the most prominent J1-M267 haplogroup in Saudi Arabia (11.6 ± 1.9 ky) is similar to that obtained previously for Yemen (11.3 ± 2) but significantly older that those estimated for Qatar (7.3 ± 1.8) and UAE (6.8 ± 1.5).

**Conclusion:**

The Y-chromosome genetic structure of the Arabian Peninsula seems to be mainly modulated by geography. The data confirm that this area has mainly been a recipient of gene flow from its African and Asian surrounding areas, probably mainly since the last Glacial maximum onwards. Although rare deep rooting lineages for Y-chromosome haplogroups E and J have been detected, the presence of more basal clades supportive of the southern exit route of modern humans to Eurasian, were not found.

## Background

The arid Arabian Peninsula can be viewed as a geographic cul de sac and passive recipient of Near East cultural and demic expansions since the Bronze Age. However, southern Arabian late Neolithic excavations revealing sorghum and date palm cultivation attest to earlier influences from East Africa and South western Asia [1]. However, after the southern dispersal route of modern humans across the Bab el Mandeb Strait was proposed [2] and further developed [3], multidisciplinary interest on this region has dramatically increased in the search for traces of such putative early southern dispersals across the Arabian threshold. From an archaeological perspective, Lower Paleolithic Oldowan industries found in the Arabian Peninsula indicated that, presumably, H. erectus or H. ergaster may have been making forays into this region [4]. There is also evidence of Middle Paleolithic Mousterian and Aterian technologies in Arabia suggesting the possibility of an expanded southern border for H. neanderthalensis and potential links with populations from Northern Africa [5]. More recent field work has sampled new Palaeolithic sites in Oman, under covering lithic assemblages with typological affinities to industries in the Levant, India and the Horn of Africa, suggesting there were a series of hunter-gathers range expansions into southern Arabia from all three refugia over the last quarter of a million years [6]. However, there are few reliable age estimates for these industries. Furthermore, the passage of these hominids to Arabia could be either through an overland route or across a waterway.

From a population genetics perspective, the most recent studies on the Arabian Peninsula have been carried out using mainly uniparental, mitochondrial DNA (mtDNA) and Y-chromosome, markers. Analysis based on maternal lineages in the region has been interpreted to represent traces of late Palaeolithic, Neolithic and more recent inputs from nearby regions into Arabia as well as signatures of authochtonous expansions [7-12]. However, the lack of deep rooting M and N sequences in the contemporary Arabian mtDNA pool leaves the proposed southern coastal route without empirical support. Although Y-chromosome basal lineages remain undetected, phylogeographic patterns indicate that the Levant was an important bidirectional corridor of human migrations [13,14]. Moreover, the Levant appeared as the main source of male lineages to the Arabian Peninsula [15]. However, Saudi Arabia, a country that occupies about 80% of the Arabian Peninsula, was not directly included. In order to fill this void, we performed a high resolution Y-chromosome SNP analysis of 157 Saudi Arabian males and a STR-based analysis of J1-M267, the most frequent Y-chromosome haplogroup in Saudi Arabia. Comparisons to other published Arabian Peninsular populations and nearby regions were made to further explore paternal traces of the modern human transit across Arabia.

## Methods

### Sample and typing

All sample collecting and genotyping tasks were performed by the Saudi Arabian collaborators of this study. Buccal swabs or peripheral blood were obtained from 157 paternally unrelated Saudi Arab males whose all known paternal lineages, at least for two generations, were of Saudi Arabia origin. Due to its moderate size we have not performed a regional subdivision of the sample. Informed consent was obtained from all the participants. This research followed the tenets of the Declaration of Helsinki. DNA was extracted using the Nucleon™ BACC Genomic DNA Extraction Kit (GE healthcare, Piscataway, NJ, USA). Sixty-seven Y-Chromosome binary genetic markers were genotyped hierarchically. Primers, polymorphic positions and haplogroup nomenclature were as recently actualized [16]. After amplification and purification, haplogroup typing was carried out in all cases by direct sequencing on the ABI 3130 *x*I Genetic Analyzer (Applied Biosystems, Foster city, CA, USA). The following 17 Y-STR loci: DYS19, DYS385 a/b, DYS389I/II, DYS390, DYS391, DYS392, DYS393, DYS437, DYS438, DYS439, DYS448, DYS456, DYS458, DYS635 and Y-GATA H4 were amplified in a Gene Am PCR System 2700 (Applied Biosystems) using the AmpF/STR Yfiler Amplification Kit (Applied Biosystems) following the manufacturers instructions. DNA fragment separation was carried out in an ABI Prism 3100 Genetic Analyzer (Applied Biosystems). STR alleles were identified by comparison to a commercial allelic ladder using Genotyper 3.7 NT software.

Our Saudi Arabia sample was compared to other Arabian Peninsula populations and to surrounding areas using data from previous studies performed at a similar level of haplogroup resolution. These samples comprise, 72 Qatari, 164 United Arab Emirate and 62 Yemeni [15]; 121 Omani and 147 Egyptian [14]; 201 Somalis [17]; 916 Lebanese [18]; 146 Jordanian [19]; 203 Iraqi (139 from Al-Zahery et al. [20] and 64 from Sanchez et al. [17]); 523 Turks [21];150 Irani [22] and 176 Pakistani [23].

### Statistical analysis

To make comparisons reliable, haplogroup frequencies were normalized to the same phylogenetic level as the sample in the published studies with the most unrefined haplogroup resolution. Analysis of molecular variance (AMOVA) and haplogroup frequency pairwise F_ST _genetic distances [24] were performed using the ARLEQUIN 2000 package [25]. Principal component (PC) analysis and two-dimensional graphics were carried out using the SPSS statistical package 11.5 (SPSS, Inc). Times to most recent common ancestor (TMRCA) for the J1-M267 clade were calculated from STR variances and by coalescence methods (see Additional File 1). Mean STR variance was estimated as proposed by Kayser and others [26] and transformed in divergence time using a mean STR mutation rate of 0.00069 per generation of 25 years [27]. For coalescence age estimations STR loci were weighted by their respective variances as described in Qamar and others [28]. Then, median-joining networks were constructed after processing the data with the reduced median network method using Network version 4.5.1.0 [29]. The rho-statistic was estimated with Network and converted into time using the above mentioned Zhivotovsky and others [27] mutation rate. For these estimations loci DYS385 were excluded and the repeats of DYS389I were subtracted to the DYS389II so that its diversity was not considered twice.

## Results

### Y-SNP analysis

**Figure 1:**
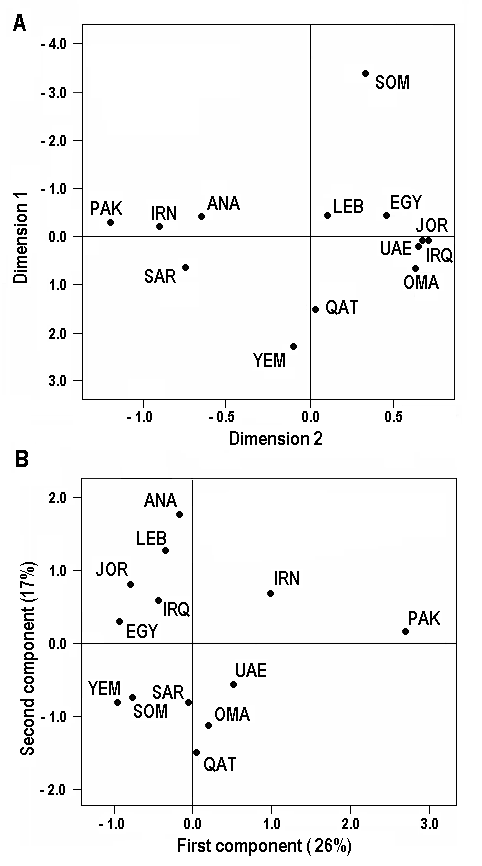
**Bidimensional plots based on Y-chromosome haplogroup frequency data in the Arabian Peninsula and adjacent populations**. **[A] **MDS analysis (stress value = 0.265); **[B] **PC analysis. Abbreviations: ANA, Anatolia; EGY, Egypt; IRN, Iran; IRQ, Iraq; JOR, Jordan; LEB, Lebanon; OMA, Oman; PAK, Pakistan; QAT, Qatar; SAR, Saudi Arabia; SOM, Somalia; UAE, United Arab Emirates; YEM, Yemen.

Only 27 of the 67 binary markers analyzed were informative in defining the haplogroup census of the Saudi Arabian sample (Table 1). The following 40 polymorphisms (M60, M130, M217, M174, P147, M33, M75, P177, P2, M215, M78, M81, M123, M329, M89, M282, M201, P20, P16, M286, M52, M197, M370, M170, M172, M410, M92, M241, M9, M20, M317, M231, M214, M119, M207, M173, M343, P25, M18 and M124) were observed at ancestral allele status in the sample set. The most abundant haplogroups in Saudi Arabia, J1-M267 (42%), J2-M172 (14%), E1-M2 (8%), R1-M17 (5%) and K2-M184 (5%) are also well represented in other Arabian populations (Table 1). When the AMOVA analysis compared all regions as unitary groups except the Arabian Peninsula, it showed a partition of variance within populations (91%) and among groups (6.8%) to be highly significant (p < 0.0001 in both cases) but an analysis among the samples within the Arabian Peninsula (2.2%) did not reach statistical significance (p = 0.10 ± 0.01). The same trend is also reflected in F_ST _based pairwise distances (Table 2), as all the distances not involving pairs of Arabian samples were statistically highly significant (p < 0.0001) but those within the Arabian Peninsula showed lower levels of significance, except when affecting Yemen (p < 0.0001) that is the most divergent population of Arabia, although showing its least distance to Qatar (p = 0.048). Precisely, in addition to Yemen, Saudi Arabia has significant haplogroup frequency differences only with Qatar (p = 0.009). Quantitatively, the main peculiarity of the Saudi male pool with respect to the rest of the Arabian samples is a significant higher presence of the J2-M172 related haplotypes (p = 0.001). On the other hand, the high divergence of Yemen is mainly due to a significant excess of J1-M267 (p < 0.0001) types, a nearly significant excess of J2-M67 types (p = 0.062), and to a lack of R1-M17 representatives (p = 0.04). In addition, southern Arabia, represented by Yemen and Oman, show a greater E1-M123 account than in northern areas (p = 0.006). As the Y-chromosome phylogeography is well structured [30,31], it is possible to roughly quantify the different male inputs from surrounding regions into Arabia. When haplogroups A, B, E-M96, E1-P2, E1-M2, and E1-M35 frequencies are assumed to be representative of the Africa contribution and frequencies of C, F, G, H, L, O, Q and R1-M17 as arrivals, across Iran, from Central, southern, and southeastern Asia, inputs of 13.4% and 16.6% from both areas are estimated for Saudi Arabia. UAE is the region with the least African (5.5%) and the greatest Iranian influence (36.8%). If the global inputs for the Arabian Peninsula are estimated to approximate 10% from sub-Saharan Africa and 22% from Iran, the remaining 68% could be considered of direct or indirect Levantine ascription. Relative relationships among the Arabian samples to nearby regions are graphically represented in bidimensional plots, resulting from MDS analysis obtained from a matrix of F_ST _distances (Figure 1a) and as the two first components of a PC analysis (Figure 1b). In the first analysis, congruently with its pair-wise distances, Somalia appears as an outlier, and the close relationship found between Qatar and Yemen is also reflected in the plot. For the rest of the Arabian samples a clear partition exists, whereas Oman and UAE appear closer to the Levant samples that include Egypt, the Saudi component positions approximates towards the northeast edge of the Near East. However, the population spatial distribution along to the first and second principal components in the PC analysis is quite different. In this case, the main haplogroups (C, H, L, O, Q and R1-M17), responsible for the positive displacement along the first component, are most abundant in Iran, Pakistan and UAE. On the opposite side are E1-M35, E1-M123 and J1-M267 that drives Yemen to the negative edge. For the second component, the main positive values correspond to the Eurasian haplogroups G-M201. I-M170, J2-M67 and R1-M269, that group all the Near East populations, including Egypt, whereas the Arabian samples and Somalia fall on the negative side pulled by their comparatively higher frequencies of the sub-Saharan Africa haplogroups E1-M2 and E2-M75 and, again, the most prevalent haplogroup in Arabia J1-M267. As the first and second principal components only capture the 26% and the 17% of the variance respectively and haplogroup E1-M78 has a minor contribution in the first and second component matrix, Somalia, that has the highest E1-M78 frequency of all the samples, does not behave as an outlier in the bidimensional PC representation. Finally, it deserves mention that, qualitatively, Saudi Arabia is also peculiar because of the presence, in low frequencies, of two underived E-M96* (1.3%) and J-M304* (1.9%) lineages that were not detected in other surveys of the Arabian Peninsula [14,15].

**Table 1:** Ychromosome haplogroup frequencies observed for Saudi Arabia and nearby regions

**Haplogroup**	**SNP**	**Sar**	**Qatar**	**UAE**	**Oman**	**Yemen**	**Somalia**	**Egypt**	**Lebanon**	**Jordan**	**Iraq**	**Anatolia**	**Iran**	**Pakistan**
A3a	M32						0.50							
A3b2*	M13				1.00			3.00		0.68		0.38		
B*	M139						1.00							
B2a*	M150	0.64	1.39											
B2a1a	M109												2.00	
B2b*	M112	1.27	1.39											
C*	RPS4Y				2.00				0.11					
C3*	M217											0.96	0.67	6.82
C5*	M356	1.27		1.23								0.38		0.57
E*	M96	1.27							0.55					
E1a*	M33							1.00	0.22					
E1b1*	P2											0.38		
E1b1a*	M2	3.82			5.00	3.23	1.50	1.00	0.66		0.99	0.19	1.33	0.57
E1b1a7*	M191	3.82	2.78	5.52	2.00			1.00						
E1b1b*	M215					1.61								
E1b1b1*	M35	2.55				3.23	1.50	3.00	0.22					
E1b1b1a*	M78		1.39	0.61	2.00		77.60	18.00	10.48	10.27	9.36	4.97	3.33	1.14
E1b1b1a2*	V13		1.39	0.61										
E1b1b1a3*	V22	0.64	1.39	6.75										
E1b1b1b*	M81			0.61			1.50	8.00	1.2	2.74		0.19		
E1b1b1c*	M123				12.00		0.50	7.00	4.26		1.48			
E1b1b1c1*	M34	4.46	1.39	3.07		8.06				13.01		5.54	0.67	
E1b1c	M329		1.39											
E2*	M75		1.39		2.00		0.50							
E2b*	M98		2.78											
F*	M89			3.68						0.68	0.49			
F1*	M282			0.61									1.33	
G*	M201				2.00		0.50	9.00	6.55		2.46	0.19	1.33	
G1*	M285	0.64		1.84								0.19	4.67	0.57
G1a	P20			0.61								0.76		
G2a*	P15	2.55	2.78	1.84		1.61				3.42		8.41	7.33	4.55
G2a1*	P16									0.68		0.96		
G2a2	M286											0.19		
G2b	M287											0.19		
G2c	M377													1.14
H*	M69	1.27		2.45	2.00									0.57
H1*	M52						0.50					0.38		5.11
H1a*	M82	0.64	1.39	1.84									2.00	
H1a1	M197													0.57
H1b	M370											0.19		
I*	M170							1.00	4.8	3.42	0.49	1.15		0.57
I1*	M253											1.15		
I2a*	P37											2.29		
I2a1	M359											0.19		
I2b*	M223											0.57		
J*	M304	1.91										0.19		
J1*	M267	40.13	58.33	34.97	38.00	72.58	2.50	19.00	20.09	30.82	31.03	8.41	11.33	3.41
J1b	M365											0.19		
J1e1	M368											0.19		
J1e2	M369											0.19		
J2*	M172				5.00		0.50	8.00		6.16	26.6	14.34		
J2a*	M410			1.23		4.84							4.00	8.52
J2a	DYS413	11.46	2.78	4.29									9.33	
J2a1	M47	2.55	1.39	1.84					15.28			1.15	3.33	
J2a2*	M67	0.64	1.39	0.61	1.00	4.84		3.00	7.75	4.79		3.63	2.00	1.14
J2a2a*	M92			1.23					0.11			2.68	1.33	
J2a5	M158											0.38		
J2a9	M339											0.19		
J2a10	M340											0.19		
J2b*	M12	1.27	1.39	1.23	4.00			1.00	2.73	2.05		0.76	2.00	
J2b2*	M241		1.39									0.96	1.33	2.27
K*	M9								0.87	0.68	0.99			0.57
K2	M184	5.10		4.91	8.00		10.40	8.00	4.69		5.91	2.49	2.67	
L*	M20				1.00				5.24		0.99	4.02		
L1	M76	1.27	2.78	2.45									2.67	
L2*	M317												0.67	
L2a*	M274												0.67	
L2b*	M349											0.19	0.67	
L3*	M357	0.64		0.61									0.67	
N*	M231											2.87		
N1c*	Tat										0.99			
N1c1*	M178											0.96		
N2*	P43												2.00	
O*	M175	0.64		0.61	1.00				0.11			0.19	0.67	
O1a*	M119			0.61										
Q*	M242	1.91		1.23	1.00			1.00	1.53			1.72		
Q1a2*	M25								0.44			0.19	4.00	
Q1a3*	M346	0.64		0.61										
L1	M76													5.11
L2*	M317													1.14
L3*	M357													6.82
O3*	M122													1.7
O3a3c*	M134													0.57
Q1a1	M120													0.57
Q1a3*	M346													1.7
Q1b	M378													1.14
R*	M207								1.31					3.41
R1*	M173			0.61	1.00			2.00			0.49	0.19	2.67	0.57
R1a*	SRY1532												0.67	
R1a1*	M17	5.10	6.94	7.36	9.00		1.00	3.00	2.51	1.37	6.90	6.88	12.67	24.43
R1a1c	M204												0.67	
R1b*	M343								0.22	13.7		0.19		
R1b1*	P25			0.61							0.99	0.38		
R1b1a	M18								0.55					
R1b1b1	M73											0.76		4.55
R1b1b2*	M269	1.91	1.39	3.68	1.00			2.00	7.31	4.11	9.85	14.53	8.00	2.84
R1b1c	M335											0.19		
R2	M124		1.39					1.00	0.22	1.37		0.96	1.33	7.39
	Sample	157	72	164	121	62	201	147	916	146	203	523	150	176

**Table 2:** Fst distances between populations. Codes as in figure 1

	**SAR**	**QAT**	**UAE**	**OMA**	**YEM**	**SOM**	**EGY**	**LEB**	**JOR**	**IRQ**	**ANA**	**IRN**	**PAK**
SAR	-												
QAT	0.023**	-											
UAE	0.004ns	0.031	-										
OMA	0.020**	0.039	0.017**	-									
YEM	0.082	0.021*	0.102	0.107	-								
SOM	0.664	0.99	0.602	0.671	1.367	-							
EGY	0.065	0.12	0.05	0.045	0.215	0.33	-						
LEB	0.057	0.108	0.045	0.053	0.188	0.326	0.028	-					
JOR	0.04	0.073	0.033	0.048	0.137	0.502	0.039	0.046	-				
IRQ	0.066	0.101	0.055	0.047	0.185	0.523	0.041	0.066	0.051	-			
ANA	0.089	0.162	0.068	0.085	0.262	0.381	0.048	0.047	0.054	0.046	-		
IRN	0.056	0.135	0.041	0.067	0.255	0.484	0.048	0.037	0.059	0.077	0.025	-	
PAK	0.126	0.21	0.095	0.116	0.352	0.539	0.093	0.086	0.113	0.128	0.055	0.029	-

* p < 0.05; ** p < 0.01; the rest of pairwise comparisons have p < 0.001 values

### Y-STR analysis

Y-chromosome STR diversity was used to obtain age estimates in Saudi Arabia for the most frequent J1-M267 haplogroup. In order to compare these estimations with other Arabian peninsula regions, we re-calculated the J1-M267 ages in UAE, Qatar and Yemen using the STR data already published for these countries [15]. STR haplotypes found in Saudi Arabia are listed in Additional file 1. Divergence times obtained using mean variance and coalescence methods are presented in Table 3. Values obtained using coalescence resulted always larger than those based on variance.

**Table 3:** Y-haplogroup J1-M267 variance and divergence times deduced from Y-STR loci

**Population**	**sample size**	**k_17_^a^**	**k_14_^b^**	**M-267 variance**	**T(ky)**	**Divergence time (ky) Mean ± SE**
UAE^c^	57	40	33	0.16	5.81	6.81 ± 1.53
Qatar^c^	42	33	28	0.19	6.71	7.27 ± 1.83
Yemen^c^	45	41	40	0.27	9.69	11.27 ± 2.03
Saudi Arabia	48	41	39	0.29	10.37	11.59 ± 1.93
**Arabian Peninsula**	**192**	**149**	**129**	**0.24**	**8.85**	**10.78 ± 1.65**

a Haplotype number using 17 Y-STR loci

b Haplotype number using 14 Y-STR loci

c Estimated from the Cadenas et al. (2007) data

## Discussion

Consistent with the rest of the Arabian Peninsula, haplogroup J is the most abundant component in Saudi Arabia embracing 58% of its Y-chromosomes. Its two main subclades (J1-M267 and J2-M172), show opposite latitudinal gradients in the Middle East. J1-M267 is more abundant in the southern areas, reaching a frequency around 73% in Yemen, whereas J2-M172 is more common in the Levant. Most probably, the significant higher presence of J2-M172 in Saudi compared to other Arabian populations is due to the larger northern boundary that Saudi Arabia shares with the Levant. The Fertile Crescent region has been considered the most probable origin of the earliest dispersions of both subclades [21,32,33]. Further subdivisions of J1-M172 have uncovered more recent Bronze age expansions from Turkey and the Balkans traced by the J2-M67/M92 and J2-M12 subgroups [21,34,35].

In a similar way, it is possible that J2-M47 signals a more recent expansion from the Levant that also affected the Arabian Peninsula. The peculiar distribution of J2-M67 in Arabia could be explained assuming maritime contacts from classical Mediterranean cultures. The presence in Saudi Arabia of three males harbouring underived J1-M304 chromosomes is intriguing. It could be that they came together with the J1-M267 or J2-M172 expansive waves, or they could represent the remnants of an old and geographically widespread Palaeolithic substrate. This type of underived chromosomes has been detected rarely in Turkey [21], in Oman and in the eastern Mediterranean area [34]. However, as the critical Levantine region has not yet been adequately dissected for J1, it seems premature to favor any of these hypotheses. The geographic pattern and most probable origin of the Y-chromosome haplogroup J in Arabia faithfully mirrors those found for the most prevalent J and R0a mtDNA haplogroups in the same region [7-9,12]. In addition, J1-M267 divergence age calculated for Saudi Arabia (11.6 ± 2 kya) and Yemen (11.3 ± 2 kya) are also very coincidental with those calculated for J1b (11.1 ± 8.4 kya) and R0a1 (9.6 ± 2.9 kya) in Saudi Arabia [7,8]. It is worth mentioning that J1-M267 ages in Saudi Arabia and Yemen are significantly older than those obtained for UAE and Qatar (Table 3)[15] and for Oman [14] pointing to a terrestrial more than to a maritime colonization. It has been suggested that Yemen could be a center of expansion for mtDNA haplogroup R0a [9].

Although the comparatively high divergence calculated for J1-M267 Y-chromosomes in Yemen [15] could be in support of primary or, at least, secondary human expansions from southern Arabia, it could also be explained as result of successive arrivals of J1 chromosomes from different source regions. Furthermore, J 1-M267 diversities found here for Saudi Arabia are of the same range than in Yemen and both smaller than the one estimated for Turkey [21]. This southwards decreasing trend is more compatible with a Neolithic arrival to Arabia via the Levant as proposed by others [15]. In fact, the coalescence ages calculated in Saudi Arabia for their most prominent mtDNA and Y-chromosome lineages merged around 10 kya. This period is coincidental with an improvement of the climatic conditions in the area that facilitated the spread of Natufian and Neolithic cultures from the Fertile Crescent north and southwards. The haplogroup E1-M123/M34 has an extended but sparse geographic distribution in Eastern Africa, the Middle East and the Mediterranean basin [14,32,35]. However, its frequency rises considerably in some populations, most probable because of isolation and genetic drift effects. This would explain frequencies as high as 11% found in some Ethiopian samples [35] or the highest (31%) found, until now, in the Dead Sea region of Jordan (Flores et al. 2005). In the Arabian Peninsula this clade is, in general, well represented but reaches significant higher frequencies in the southern countries of Yemen and Oman compared with northern areas. An East African origin, and posterior spread to the Near East through the Levantine corridor, of this lineage was proposed based on the much larger variance of this clade in Egypt (0.5) versus Oman (0.14) [14]. On the contrary, other authors have suggested that E1-M123 may have originated in the Near East because of its generalized implantation there [21] compared to its presence in Eastern Africa, mainly just localized in Ethiopia [32]. Recent E-M123 haplogroup variances calculated for Yemen (0.14) and UAE (0.25) were also lower than the Egyptian one [15]. In addition, haplotypic differences found for those two Arabian countries indicated that they do not share a common ancestry [15]. So, from an Arabian Peninsula perspective, E1-M123 could have come from Ethiopia, across the Horn of Africa, or from the Levant, or even from both sources, forming independent isolates. Global male inputs from Sub-Saharan Africa and Asia across Iran, not the Levant, into the Arabian Peninsula have been estimated in this study, as 13.4% and 16.6% from both source areas respectively. Recent mtDNA studies on the same Arabian Peninsula countries [7-9,12] have confirmed a notable female-driven sub-Saharan African input with a mean value around 15% for all the Peninsula, although frequencies as high as 60% have been detected in Hadramawt populations of Yemen [9]. Curiously, the Iranian female flow (18%) was also rather similar to that calculated for Africa. Although a slight ratio excess of Sub-Saharan African female versus male gene flow is detected (1.12) we do not found the strong sexual bias proposed by other authors for Arabian populations and attributed to the peculiarities of the recent slave-trade [12,36]. Without dismissing the role mediated by slavery, the geographical distribution of these sub-Saharan African lineages in the Arabian Peninsula seems to indicate a prehistoric entrance of a noticeable portion of these lineages that participated in the building of the primitive Arabian population [8,9]. The presence of two underived E-M96 Saudi lineages raises interesting questions related to the macrohaplogroup DE-YAP phylogeography. The recent resolutions of the CDEF-M168 tripartite structure to the bipartite DE-YAP and CF-P143 [16,31] extends the conversation regarding the early successful colonization of Eurasia. While several scenarios remain potentially possible the most parsimonious model is the most prudent. This model proposes the successful colonization of Eurasia by migration(s) of populations containing precursor Y-chromosome founder macrohaplogroup CDET-M168 and basal mtDNA L3 representatives. Regions near but external to northeast Africa, like the Levant or the southern Arabian Peninsula could have served as an incubator for the early diversification of non-African uniparental haplogroup varieties like Y chromosome DE-YAP*, CF-P143* and mtDNA M and N molecular ancestors. These would have spread globally and diversified over time and space. This model would imply that both CF-P143 and the DE-YAP evolved nearby but outside Africa. One DE-YAP* ancestor would have spread to Asia and evolved to haplogroup D while another DE-YAP* returned to northeast Africa and evolved into hg E. It is noteworthy that DE-YAP* has been detected at low frequency in Africa [37]. Again, this hypothesis has its mtDNA counterpart as it is well documented that, in the Palaeolithic, at least three clades (X1, U6, M1) derived respectively from the three main Eurasian macrohaplogroups (N, R, M) came back to North Africa from Asia [38-42].

Within this frame, it should be expected that E-M96* types appear in Africa although its presence in the Arabian Peninsula instead Eastern Africa would not compromise the last proposed model. It could be suggested that these E-M96 Saudi lineages have a sub-Saharan Africa ancestry. However, at least for one of them, all their known male ancestors belong to a big Shammar Arab tribe that ruled much of central and northern Arabia from Riyadh to the frontiers of Syria and northern Iraq. In addition, it might be present in Lebanon [18]. However, as the authors did not type more markers derived within the E-M96 background such as P147, P177, P2, P75 or M329, more comprehensive phylogenetic resolution of YAP derived Y-chromosomes in the Middle East and North and East Africa are necessary to explore the topic further. In any case, the presence of E-M96* in Saudi Arabia should not be taken as support of the southern exit of modern humans across the Srait of Bab el Mandab as no D nor CF underived lineages have been yet found in this area.

## Conclusion

The Y-chromosome genetic structure of the Arabian Peninsula seems to be mainly modulated by geography. The data confirm that this area has mainly been a recipient of gene flow from its African and Asian surrounding areas, probably mainly since the last Glacial maximum onwards. Although rare deep rooting lineages for Y chromosome haplogroups E and J have been detected, the presence of more basal clades supportive of the southern exit route of modern humans to Eurasian, were not found.

## Authors' contributions

KKA and AH was in charge of carrying out the sequences, collection of samples, haplogrouping and writing part of the manuscript. AMG, JML, VMC and PAU were in charge of design of the experiments, analysis of the data and writing manuscript. All authors read and approved the final version of the manuscript.

## Supplementary Material

Additional file 1 **Table S1**. Y-chromosome STR haplotypes for haplogroup J1-M267 in Saudi Arabia. Click here for file
